# Emerging and reemerging forms of *Trypanosoma cruzi* transmission

**DOI:** 10.1590/0074-02760210033

**Published:** 2022-05-16

**Authors:** Maria Aparecida Shikanai Yasuda

**Affiliations:** 1 Universidade de São Paulo, Faculdade de Medicina, Departamento de Moléstias Infecciosas e Ptarasitárias, São Paulo, SP, Brasil; 2 Universidade de São Paulo, Hospital das Clínicas da Faculdade de Medicina, Laboratório de Imunologia, São Paulo, SP, Brasil; 3 WHO Technical Group IVb on Prevention and Control of Transmission and Case Management of Trypanosoma cruzi Infections, WHO, Geneva, Switzerland

**Keywords:** Chagas disease, *Trypanosoma cruzi* infection, vertical, blood transfusion, organ transplant and oral transmission, emerging and reemerging forms of Chagas disease

## Abstract

This review aims to update and discuss the main challenges in controlling emergent and reemergent forms of *Trypanosoma cruzi* transmission through organ transplantation, blood products and vertical transmission in endemic and non-endemic areas as well as emergent forms of transmission in endemic countries through contaminated food, currently representing the major cause of acute illness in several countries. As a neglected tropical disease potentially controllable with a major impact on morbimortality and socioeconomic aspects, Chagas disease (CD) was approved at the WHO global plan to interrupt four transmission routes by 2030 (vector/blood transfusion/organ transplant/congenital). Implementation of universal or target screening for CD are highly recommended in blood banks of non-endemic regions; in organ transplants donors in endemic/non-endemic areas as well as in women at risk from endemic areas (reproductive age women/pregnant women-respective babies). Moreover, main challenges for surveillance are the application of molecular methods for identification of infected babies, donor transmitted infection and of live parasites in the food. In addition, the systematic recording of acute/non-acute cases and transmission sources is crucial to establish databases for control and surveillance purposes. Remarkably, antiparasitic treatment of infected reproductive age women and infected babies is essential for the elimination of congenital CD by 2030.

Chagas disease (CD) is an active disease in urban centres of endemic and non-endemic areas in several continents due to immigration of infected population to the urban centres of endemic regions and non-endemic areas (Europe and Asia, Australia, US and Canada, Japan, Australia,^1^ affecting according to estimates in 2006, around 6-7 million persons in Mexico and Central and South America and causing 12,500 deaths per year and 41,200 new cases annually.^2^

Although vector and blood transmitted disease have been reported under control in almost all endemic areas, the globalisation of the disease in urban centres of all continents made possible the reemergence of blood and/or organs transplant and maternofetal transmission in the US,^3^ ^,^ ^4^ ^,^ ^5^ ^,^ ^6^ Europe, Australia, Asia,^1^ ^,^ ^7^ ^,^ ^8^ ^,^ ^9^ ^,^ ^10^ the latter two still as challenges in endemic regions.^11^ ^,^ ^12^ Additionally, regarding vector control, autochthonous chronic CD cases have been reported in previously non registered endemic areas such as part of Amazonia and non-endemic regions US.^6^ ^,^ ^13^ ^,^ ^14^

*Trypanosoma cruzi* infection prevalence in immigrants was variable from 1-26%, depending on the country and nationality.^1^ ^,^ ^7^ Approximately 300,000 individuals are estimated to be infected with *T. cruzi* in the US^15^ ^,^ ^16^ and around 97,556 in Europe.^17^ Different initiatives have been implemented in several countries of non-endemicity aiming to control blood transfusion and organs transplant transmission. However, other factors, as the physician unawareness of vertical transmission and congenital CD (cCD) reactivation are unfavorable for their control and prevention.^18^ ^,^ ^19^

Of note, acute CD (ACD) emerged in unpredictable circumstances in the Brazilian Amazon region and other Latin American areas, where the domiciliary triatomine cycle has been under control, transmitted by contaminated food as a result of serious disturbances in the wild cycle of vectors and reservoirs of *T. cruzi*.^20^ ^,^ ^21^

As one of the neglected tropical disease potentially controllable with a major impact on morbimortality and socioeconomic aspects, CD has been included in the World Health Organization (WHO) global plan to interrupt four transmission routes by 2030 (vectorial, blood transfusion, organ transplant and maternofetal).^22^ The implementation of the World Chagas Day during the 72nd World Health Assembly (Geneva/Switzerland, 2019) provides not only more visibility but also contributes to establish comprehensive integral health care with diagnosis, treatment and quality of life for millions of infected patients.^23^

The aim of this work is to discuss the main challenges in the control of emerging and reemerging transmission routes through blood transfusion, organ transplantation, maternofetal and contaminated food.

**Transmission by blood or blood products**

Tables I ^24^ ^,^ ^25^ and II ^8^ ^,^ ^17^ ^,^ ^26^ ^,^ ^27^ show the number of infected people as well as prevalence estimates of vectorial and congenital transmission and of blood bank candidates in endemic and non-endemic countries.

**TABLE I:** Distribution of *Trypanosoma cruzi* infected people and reproductive age women and estimates of vectorial, maternofetal transmission, and prevalence in blood bank candidates in regions of endemicity^*a*^

Country	*T. cruzi* infected people	Annual number of vectorial transmissions	Annual number of congenital infections	Number of 15-44-year-old women	Prevalence (%) in the blood bank candidates
Argentina	1,505,235	1,078	1,457	211,102	3.13
Bolivia	607,186	8,087	616	199,351	2.32
Brazil	1,156,821	46	571^*b*^	119,298	0.18
Chile	119,660	0	115	11,171	0.16
Colombia	437,960	5,274	1046	116,221	0.41
Costa Rica	7,667	10	61	1,728	0.045
Ecuador	199,872	2,042	696	62, 898	0.19
El Salvador	90,222	972	234	18,211	1.610
Guatemala	166,667	1,275	164	32,759	1.34
French Guiana-Suriname	12,600	280	18	3,818	NA
Honduras	73,333	933	257	16,149	0.126
Mexico	876,458	6,135	1,788	185,600	0.089
Nicaragua	29,300	383	138	5,822	0.124
Panama	18,337	175	40	6,332	0.056
Paraguay	184,669	297	525	63,385	0.34
Peru	127,282	2,055	232	28,132	0.038
Uruguay	7,852	0	2	1,858	0.040
Venezuela	193,339	873	665	40,223	0.110
Subtotal	5,742,167	29,925	8,668	1,124,930	0.089

*a*: Source: WHO^24^; *b*: add more 543 RN from Bolivian immigrants in Brazil, according to the estimates of Luna et al.^25^

In endemic countries, universal screening should be implemented in every blood bank candidate by applying a reliable and reproducible high performance serological test with about 99-100%, followed by confirmatory test with at least 95% of specificity.^28^

It is possible to observe that in some regions of non-endemicity, the prevalence is similar or higher than those of endemic areas, perhaps by concentration of immigrants (Table II).^8^ ^,^ ^17^ ^,^ ^26^ Additionally, high underdiagnosis indexes between 89% and 99% of expected cases are estimated in Europe^26^ and risk of blood transfusion transmission has been recognised in several countries (US, Spain, Canada, Belgium),^8^ ^,^ ^9^ ^,^ ^29^ leading to the implementation of different policies in some regions of non-endemicity, as observed in Table III.

**TABLE II:** Distribution of *Trypanosoma cruzi* infected people in regions of non-endemicity and estimates of annual number of congenital infections, of infected pregnant women and prevalence in blood bank candidates

Country	*T. cruzi*	Infected people	Annual number of congenital infections^(26)^	Infected pregnant women^(26)^	Prevalence (%) in blood bank Angheben et al.^(8)^
	Strasen et al.^(17)^	Basile et al.^*(26),a*^			
Spain	75,358.53	47,984-86,618	16-162	1,125-2,226	1/218
Italy	9,200.20	6,464-12,036	1-6	55-76	3.9/100
Germany	2,007.97	1,123-1,481	NR	NR	
France	2,065.98	2,148-2,823	1-5	53-74	1/32,800
Netherlands	1,885.73	967-1,773	NR	NR	
Portugal	1,548.96	1,255	1-3	40	
United Kingdom	1,507.22	6,111-12,201	58-84	54-84	1/12,861
Sweden	1,302.64				
Switzerland	1,171.48	1,584-3,971	0-1	6-8	
Belgium	683-921	683-921	0-1	10-13	
Europe	97,556	68,318-123,078	20-184^e^	1,347-2,521	
US	238,091^(16)^ - 326,000				1/27,500
Canada	100,000^(8)^				3.1/10^5^
Japan	4,000^(10)^				
Australia	1,928^(27)^				0.04%^*(27),b*^
New Zealand	82^(27)^				

*a*: high underdiagnosis index, estimated to be in Europe between 89% and 99% of expected cases^(26)^; *b*: Australian Red Blood Service, 2010 - No systematic screening.

**TABLE III:** Health policy for blood donations (transplantation)^*a*^ and year of implementation

	Universal screening	Screening (at risk donors)^*b*^	Deferral (at risk donors)	Exclusion (infected donors)	No specific measures	Transfusion transmission
US	X					X
Canada		X				X
UK		X (X)				
Spain	(X)	X (X)				X
France	(X)	X				
Switzerland		X				
Italy		in process 2014 (X)				
Belgium		X				
Portugal		No available^*c*^	X (X)			
Sweden		X	X^*d*^			
Other European countries		X (X) EU guidelines		X^*e*^ (X^*e*^ hearts, intestines)		
Australia/New Zealand			X^*f*^			X
Japan			X			
China					X	

*a*: (X) - in parenthesis - refers to transplantation; *b*: at risk: born in endemic regions, or born to mothers native of endemic regions, or recipients of blood transfusions in endemic regions. Canada and US include persons who lived at least 6 months or 3 months, respectively, in endemic areas and Council of Europe (Guide to the preparation, use and quality assurance of blood components-20th edition, 2020 and Guide to the quality and safety of Organs for transplantation 7th Edition, 2018) recommends at risk donors screening; *c*: 2015 - No available data; *d*: exclusion of at risk donors who lived more than five years in Chagas disease endemic countries (no information about those exposed and not tested yet); *e*: other European countries follow European Commission’s directives 2004/33/CE and 2006/17/CE, 2018/7th Ed/ CE; *f*: epidemiological form excludes at risk candidates; no screening test for at risk donors and blood derivates are only prepared if serology had been negative.^(27)^

In the US blood banks, screening is universal and in Canada, screening targets people at risk for CD. Seven countries in Europe, France, Italy, Portugal, Spain, Sweden, Switzerland, and the United Kingdom, where the majority of Latin American immigrants live currently, have implemented or are recently changing their recommendation for screening at blood donor with risk factors for *T. cruzi* infection.^30^ Target screening does not cover donors born to mothers, who lived in endemic areas or donors that lived less than five years in endemic areas,^31^ neither donors that travelled during three months to vector transmission area, as registered in infected people in the US.^31^

In parallel, the deferral of donation for six months after travelling to endemic regions, applicable for some diseases is not useful for CD, since chronic disease occurs after the infection without symptoms in the majority of cases, so the disease could be not easily suspected in blood/organ donors’ candidates. Moreover, even with recommendation for screening people at risk guided by European guidelines, it is not known whether these measures have been implemented in some countries.^30^

In addition, countries in the Western Pacific region do not apply any health policy to systematically identify individuals at risk for *T. cruzi* infection. In Japan, deferral of at risk donor and in China no policies have been described. In Australia, screening is carried out using a questionnaire to identify at-risk donors through clinical and epidemiological data and exclusion of those affected.^27^

*Comments* - Considering the current situation in regions of endemicity and non-endemicity it is strongly recommended: (a) universal screening in regions of endemicity or non-endemicity with a high concentration of infected immigrants through a highly sensitivity serological method in blood banks followed by confirmation by a high specific method, according to the criteria recommended by Pan American Health Organization (PAHO);^28^ (b) in regions of non-endemicity, with a low prevalence of infected immigrants, screening target at risk people, including old or recent infection, followed by laboratorial confirmation (vectorial infection during the life or during travel to endemic areas, blood/blood products/organ transplant, congenital disease); (c) blood products from these donors should not be used except after exclusion of *T. cruzi* infection. Epidemiological data should consider the region where the donor lived, the presence of the vector in the house or in the neighborhood and/or relatives and mother with CD,^32^ even in the absence of signals and symptoms, characteristics of the chronic indeterminate or early cardiac phase of CD.

**Solid organ transplantation (SOT) and allogeneic haematopoietic stem cells transplantation (Allo-SCT) transmission**

As a route of CD transmission known in endemic regions since the 80s,^33^ SOT emerged in urban centres of endemic and non-endemic regions with high impact due to immunodepressed receptor evolving with serious illness.^4^ Increasing number of solid transplants have been reported due to improved immunosuppressive management as well physician’s awareness and knowledge since the first publications.^33^ Although less frequently, Allo-SCT has also been described in endemic areas.^34^

In contrast to retrospective studies of donor derived infections where the infected recipients were suspected lately, when clinical symptoms appear and the prognosis is worse, active monitoring by parasitological and molecular methods allows the detection of asymptomatic infections, introduction of early treatment and better prognosis.^11^ ^,^ ^35^ The risk of potentially fatal outcomes in immunosuppressed recipients, specially from heart and kidney-pancreas transplants under aggressive therapy for graft rejection, requires prevention strategies starting with donor screening.

Universal screening is highly recommended in endemic areas and/or non-endemic regions with Latin America immigrants’ prevalence (Table III).^30^ In the US, only 19% of organ procurement organisation performed universal or targeted donor screening for *T. cruzi* infection by 2009.^36^ Target screening has been recommended when the potential donor or recipient has been in risk areas for acquiring of *T. cruzi* infection during at least three months^29^ or was born in endemic areas (Table I).

Guidelines for organs transplant for prevention and management of CD were recorded in Argentina,^37^ Europe (including South American experts)^38^ and US^39^ guiding for serological screening but not always recommending high (99-100%) sensitivity tests.^28^ Usually, one screening test is employed according to the choice of responsible institution, followed by one confirmatory test for CD diagnosis. In Brazil, although regulated by National Transplant Agency, the main challenge is to consider the use of highly sensitive and specific test as mandatory regardless of the competitive cost at each centre. In fact, a few cases have been reported of suspected donor derived infection in a liver and in a renal transplant recipient associated with a single negative serological test in the donor.^40^ ^,^ ^41^ Moreover, considering the safety and effectiveness of screening in this first phase, we recommend the employment of two highly sensitive tests as strategy for donor screening, followed by a high specific confirmatory test, as recommended by PAHO, 2019.^28^

Donor status concerning *T. cruzi* infection should be known before the transplant. In case of confirmed infection, the next step is to decide whether the donor should be treated before the procedure (Figure). In this situation, 60 days and, at least 30 days is the minimum period recommended instead of a shorter period. In case of emergency or retrospective knowledge of the donor’s infection, CD monitoring and /or prophylaxis of non-immune recipient is recommended.

**Figure:**
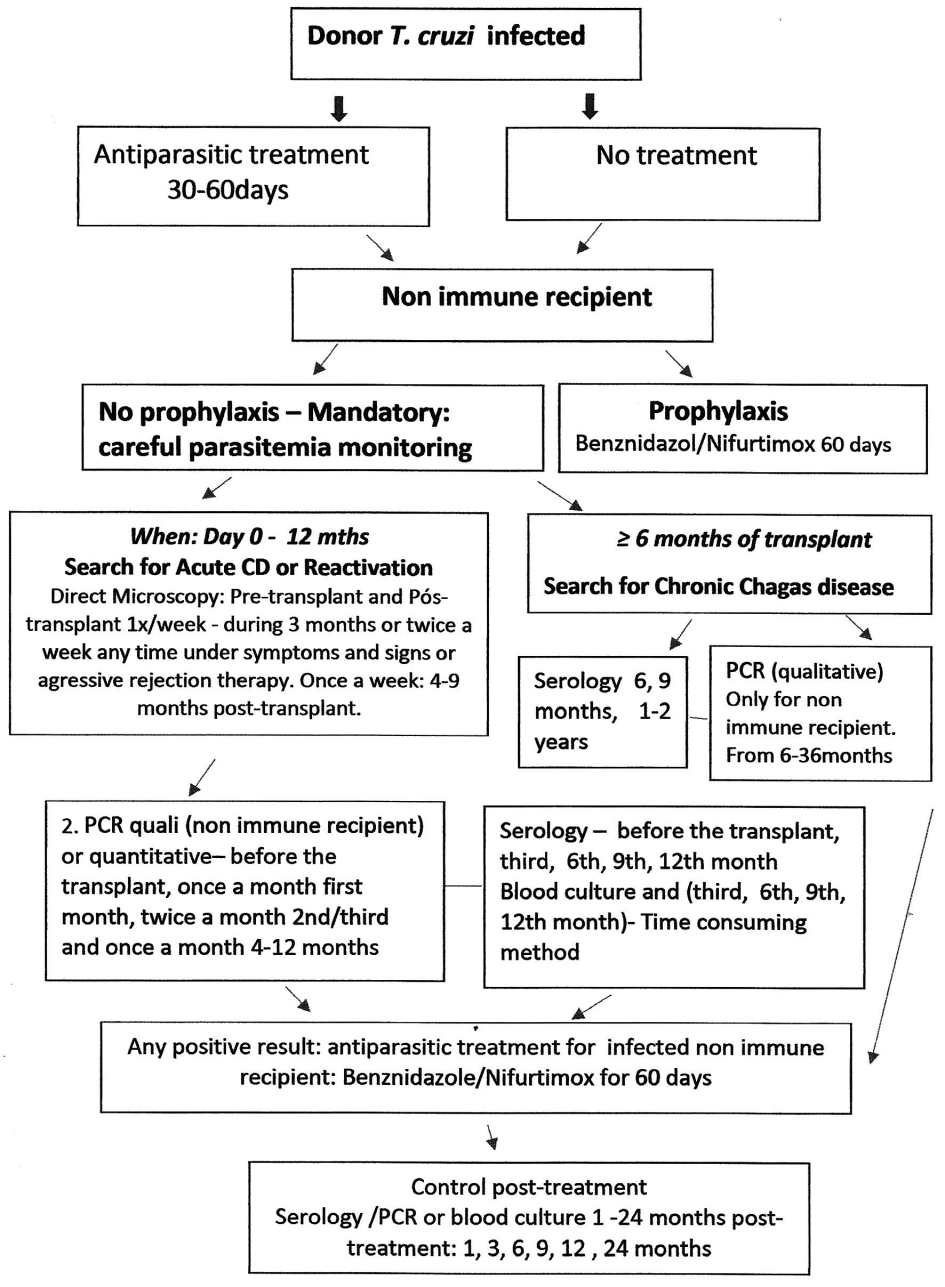
Algorithm for *Trypanosoma cruzi* infected donor and transplant recipient.

Regardless of any decision, careful monitoring of recipient parasitaemia is mandatory, starting before the transplant and considering the patient’s immune status after the procedure.

It is essential in the follow-up:

A - To search for ACD during immunosuppression (and/or reactivation) in the initial months post-transplant and/or under aggressive immunosuppressive therapy for graft rejection or under signs and symptoms of suspected ACD or reactivation:

(i) Methods? Direct parasitological tests in peripheral blood or other secretions, preferably through microscopy concentration methods (microhematocrit, Strout, buffy coat) more sensitive than stained slides or fresh blood are recommended. Qualitative polymerase chain reaction (PCR) is highly sensitive and recommended for diagnosis of the primary infection since the initial days post-transplant up to 12 months, although not advisable to distinguish CD from reactivation, for which quantitative PCR is indicated. Indirect parasitological methods complement CD diagnosis but they are time consuming and less safe than PCR.

(ii) When? Day 0 to 12 months after the transplant, according to the method:

Direct microscopy - After the transplant: once a week durCongenital Chagas disease (cCD)ing three months and twice a week any time under symptoms/signs under suspicion of reactivation or during aggressive immunosuppressive therapy for graft during the follow up period. Once a week from 4-9 months, when the majority of acute cases where diagnosed.

PCR - 0, once a week in the first month, twice a month int he 2nd and third month and once a month from 4-12 months.

Other indirect enrichment parasitological methods (blood culture, xenodiagnosis): Day 0, 15 days, one month after the transplant up to 6-12 months. Repeat each 3 months: 3, 6, 9 and 12 months after the transplant.

Serology - 0, 3 weeks, 1,3,6, 9, 12 months. This method is less sensitive in transplanted patients than in immunocompetent patients, since T dependent antibodies production is impaired as seen by negative seroconversion in 43.4% of infected renal recipients in Argentina.^11^

(iii) How to interpretate immediate positive and negative results post-treatment? Persistent parasite identification by microscope examination raises the possibility of therapeutic failure, recommending the change of the antiparasitic drug. On the other hand, negative results should be carefully interpretated since these drugs temporarily inhibit the parasite and parasitaemia could return later in the follow-up. After 3-6 months of treatment, PCR and other parasitological methods are more reliable for parasitaemia monitoring than direct microscopy, excluding recurrence episodes.

B - To search also for chronic Chagas disease, asymptomatic or symptomatic, acquired by transplant in the late post-transplant phase, from 6 months post-transplant:

(i) Methods? PCR and serology are recommended. The latter is less sensitive in immunosuppressed patients as cited before^11^ and should be repeated many times if negative and never employed as unique method. If possible, parasitological enrichment methods (blood culture, xenodiagnosis) are useful for the identification of phenotypic and genotypic characteristics of the parasite.

(ii) When? Preferably, collect samples before the transplant. In the absence of antiparasitic treatment: repeat 6, 9, 12, 18, 24 and 36 months after the transplant. During antiparasitic treatment and even a few periods later, negative results for indirect parasitology/serology/PCR do not represent therapeutic success, as cited before and this negative result should remain for a long period to indicate such effect.

In conclusion for diagnosis of ACD, direct concentration parasitological methods (Strout, buffy coat, microhematocrit) together PCR are recommended for the entire period of immunosuppression, mainly in the first 9-12 months after the transplant. In case of chronic CD, this follow-up should continue at least 36 months by PCR and serology preferably, and, if accessible, by parasitological enrichment method (haemoculture). After antiparasitic treatment, follow-up up to 12-36 months after the end of therapy.

Table IV depicts case reports and transmission rates from donors to receptors associated with prophylaxis (0%) with no accurate cure control and without prophylaxis (15.5%) in Kidney transplant.^11^ ^,^ ^33^ ^,^ ^35^ ^,^ ^42^ ^,^ ^43^ ^,^ ^44^ ^,^ ^45^ ^,^ ^46^ ^,^ ^47^ ^,^ ^48^ In liver transplantation, 25.9% and 14.3%, respectively with or without prophylaxis,^35^ ^,^ ^47^ ^,^ ^49^ ^,^ ^50^ ^,^ ^51^ ^,^ ^52^ ^,^ ^53^ ^,^ ^54^ again accurate methods for cure control are sometimes incompletely employed. As indicated in Table IV, no accurate control means that serology is not sufficient as the unique method for therapeutic control in immunosuppressed recipients. Additionally, indirect parasitological/molecular methods are complementary for chronic CD in the late and/or short follow-up period. The same table depicts transmission rates in a few cases of heart, lung transplant, stem cell and kidney-pancreas transplantation with or without prophylaxis.^35^ ^,^ ^36^ ^,^ ^48^

**TABLE IV:** Donor derived infection by kidney, liver, heart and hematopoietic stem cell transplants

Authors kidney	Donor + (N)^*a*^	% infected organ /total receptor^*a*^	Diagnosis	Infection diagnosis: weeks	Follow up^*b*^ (months)	Prophylaxis	Death^*c*^
Chocair^(33)^ 1981, BR	4	Case series	Buffy coat^*d*^	4,12,16,62	No reported	No	No
Figueiredo^(42)^ 1990, BR	1	Case report	Microscopy^*d*^	22	21	No	No
Cantarovich^(43)^ 1992, ARG	26	3/26	Parasitaemia/clinical	2,6.5,35	“long term evolution”	No	No
Riarte^(11)^ 1981 ARG	16	3/16	Strout, culture	5, 8, 23	12-60	No	No
De Arteaga^(44)^ 1992, ARG	6 cadaveric 1 live	0/7	Parasitaemia, serology	Non detected	Non accessible	No	No
Vásquez^(45)^ 1992, ARG	8 cadaveric 1 live	2/9	Strout, micro-haematocrit, serology^*d*^	5-9	4^*b*^ -36	No	No^c^
Sousa^(46)^ 2009, BR	9+ Serology	0/9	Serology^*d*^	Non detected	3^*b,d*^ , 64-123^d^	Recipient 14 days BNZ PT	No
Huprikar^(35)^ 2013, US	10 patients (17 organs)	3/17 (1†KP)	Clinical/blood culture /PCR	5,7, 17	1.8^*†*^ , 3-4^*d*^ , 14, 24-57	No	1 Kidney pancreas
Cura^(47)^ 2013, ARG	1+/4	1/4 (0 KP)	PCR, strout, serology	13	14-30	No	No
Cicora^(48)^ 2017, ARG	6+/73 (8.2%)	0/6	Thick-blood^*d*^ film technique	No	7^ɫ^-60	Donors 30days BNZ pre-transplant	No
Total Kidney Prophylaxis No Prophylaxi^s^		12/93=12.9% 0^b^/15 = 0% 12/78 = 15.5%				Prophylaxis: 6 donors and 9 recipients	
Authors liver	Infected donor	% infected organ /total receptor^*a*^	Diagnosis	Infection diagnosis: weeks	Follow up^*b*^ (months)	Prophylaxis	Death^*c*^
D´Albuquerque^(49)^ 2007, BR	4	0/4	Serology^*d*^ 3,6,12m Microscopy^*d*^ 3,6 m	Non detected	12^*b*^	BNZ PT	No
Salvador^(50)^ 2011, Spain	2	1+^*e*^ /2	PCR/serology	8-36 Elisa +	8^*b*^ -36	BNZ PT	0
Goldaracena^(51)^ 2012 ARG	1 HIV	0 case report	Strout, PCR, serology	Non detected	2^*b*^	No	No
McCormack^(52)^ 2012, US	9	2/9= 22%	Parasitaemia Strout	12-16	15^*b*^	No	No
Huprikar^(35)^ 2013, US	12	2/11 = 22% No prophylaxis 0/1 Prophylaxis	Clinical/PCR/ blood culture	8, 12	2^*b*^ , 3^*b*^ , 7-10^*b*^ , 53	9 No 1 Yes BNZ PT	No
Cura^*(47),a*^ 2013, ARG	3	3/3	PCR, strout, serology	5, 6, 14	17-23	No	No
Rodrigues-Guardado^(53)^ 2015, ARG	1	0 case report	Serology micro-haematocrit, PCR	44	64	No	No
Beldarramo^(54)^ 2017, ARG	4+/102 (3.92%)	0/4	qPCR	Non detected	Median: 54	No	No
Total liver Prophylaxis No prophylaxi^s^ e		8/34 = 23.5% 1/7 = 14.3% 7/27 = 25.9%				Prophylaxis 7 receptors	
Authors lung	Donor + (N)	% infected organ /total receptor^*a*^	Diagnosis	Infection diagnosis: weeks	Follow up (months)^*b*^	Prophylaxis	Death^*c*^
Cura^(47)^ 2013, ARG	1	1/1	Strout, PCR	10	8	No	No
Huprikar^(35)^ 2013, US	1	Bilateral lung	PCR, blood culture, serology	29	7	No	No
Salvador^(50)^ Spain, 2017	1	0/1	qPCR, serology	Non detected	11	BNZ PT	No
Authors heart	Donor + (N)^a^	% infected organ /total receptor^*a*^	Diagnosis	Infection diagnosis: weeks	Follow up (months)^*b*^	Prophylaxis	Death^*c*^
Huprikar^(35)^ 2013, US	3+/4^*f*^	3/4	Smear, PCR, blood culture, serology	3,7,9	2, 7, 9	No	1
Allogeneic SCT author	Donor + (N)^*a*^	% infected organ /total receptor^*a*^	Diagnosis	Infection diagnosis: weeks	Follow up (months)	Prophylaxis	Death^*c*^
Altclas^(34)^ 2011, ARG	3	0/4	0 (strout, parasitology, serology)	Non detected	3, 4, 11	No-1 patient 2 BNZ-30 d	No

BR: Brazil,: ARG: Argentina; BNZ PT: benznidazole in the immediate post-transplant period for 60 days, except when indicated; *a*: comparative infection rates only for cases’ series with post-transplant follow-up period; *b*: control of antiparasitic treatment in transplants need to be extended up to 24 months by parasitological methods or polymerase chain reaction (PCR) repeatedly together serology. *c*: deaths related only to Chagas disease (CD); *d*: PCR or parasitological tests for chronic CD were not employed for diagnosis of chronic CD after 3 months; *e*: positive serology means chronic CD even though asymptomatic; *f*: a 4th recipient from a non-immigrant donor with inconclusive serology (Elisa + by commercial tests and negative by IFA at CDC) did not have detectable anti-*T. cruzi* antibodies after the transplant, but donor infection could not be proved.

Table IV shows only two deaths by CD^35^ in highly immunosuppressed heart and kidney recipients with delayed ACD diagnosis by unknown donor infection. An additional question, is the use of infected donors advocated by some authors in liver and kidney^48^ ^,^ ^50^ ^,^ ^53^ and even stem cell transplants since antiparasitic donor treatment is ensured, followed by recipient’s prophylaxis and careful parasitaemia control. As discussed, risk of chronic CD is not always excluded. This circumstance represents an exception, reserved only for individual emergency situation and/or joint decision of health committees of professionals and patients’ communities waiting for transplants where the lack of organs and long queues are associated with significant mortality.

*Comments* - Universal or target screening for organs transplant donor should be mandatory, and at least two high performance serological tests are recommended. Serological, molecular and parasitological methods should be employed repeatedly, according to different stages of the transplant, and the recipient’s immunological “status”. Monitoring with quantitative PCR (qPCR) should be implemented to support an adequate parasitaemia control with or without recipient prophylaxis and/or donor treatment. Evidences of the benefit of prophylaxis need to be confirmed in larger samples carefully monitored for longer periods through adequate methods for ACD and chronic CD diagnoses.

**Congenital Chagas disease (cCD)**

Maternofetal represents the main route of *T. cruzi* transmission in free vector regions within and outside Latin America over blood products and organ transplants transmissions. Its interruption around 2030 has been decided as a goal for WHO in 2018,^55^ after demonstration of its prevention in several reports.^56^ ^,^ ^57^ ^,^ ^58^ ^,^ ^59^ ^,^ ^60^

A systematic review including 13 case reports/series and 51 observational studies^61^ estimated the pooled congenital transmission rate as 4.7% [95% confidence interval (CI): 3.9-5.6%], higher in endemic than in non-endemic areas (5.0% vs. 2.7%). Congenital infection rates depend on design and period of study, methods for diagnosis, patients´ age, region and presence of the vector transmission as well as patients’ age. Most reports are from Latin American, Southern Cone and Bolivia; rates are less known in Mexico and Central America. It is estimated in 6.1% in Argentina,^62^ ^,^ ^63^ 5.0-6.0% in Bolivia,^64^ 0.8-6.3% in Mexico,^63^ ^,^ ^64^ ^,^ ^65^ 3-10% in Paraguay,^66^ 1.8% in Chile,^67^ 1.7% in Brazil^68^ and 0% in Honduras.^63^ The estimated numbers of infected pregnant women and newborns are 40,000 and 2,000 newborns in Canada, Mexico and the United States.^62^ cCD was reported in several continents and the estimated annual numbers of children congenitally infected are represented in Tables I-II.^5^ ^,^ ^10^ ^,^ ^69^ ^,^ ^70^

**Determinants of congenital transmission**

*cCD and temporal evolution* - The spectrum of cCD is variable from abortions and stillbirths, hydrops foetalis congenital megaesophagus prematurity and low birth weight in the early stage of pregnancy to severe cases similar to sepsis in late intrauterine or perinatal infection.^71^ ^,^ ^72^ ^,^ ^73^ ^,^ ^74^ The last group is described as TORCH: toxoplasmosis, *Treponema pallidum*, rubella, cytomegalovirus, herpesvirus, hepatitis and human immunodeficiency virus, parvovirus B19, and enteroviruses^75^ with hepato-splenomegaly and/or anaemia and/or thrombocytopenia and, less frequently, meningoencephalitis and/or myocarditis, pneumonitis.^76^ ^,^ ^77^ Other have mild symptoms and signs (hepatomegaly, hepatosplenomegaly) or are asymptomatic. The majority of infected pregnant women, asymptomatic, may be at increased risk of cCD transmission.

Rates between 35.0-68.4% of symptomatic cases have been reported in different countries and/or periods.^64^ ^,^ ^76^ ^,^ ^77^ In a Bolivian cohort, such rate decreased from 50% to 18% and mortality from 20% to 4%, in 1992-1994 and 1999-2001, respectively.^64^ Asymptomatic infection seems to be more frequent than severe cases, recently,^78^ possibly due to the vector control and to better prenatal/neonatal care. In parallel, rates of infected women decreased from 28% to 17% but transmission rate of cCD was similar (5-6%) in both periods.

*Maternal parasitaemia* - Higher morbidity and mortality of cCD was associated with higher parasitaemia in mothers living in high vector density areas compared to those living in vector free areas.^78^ ^,^ ^79^ Of note, influence of high maternal parasitaemia on the transmission rates over 50% have been shown in *T. cruzi*/HIV infected mothers without highly active antiretroviral therapy (HAART) control.^18^ ^,^ ^76^ ^,^ ^80^ ^,^ ^81^ ^,^ ^82^ ^,^ ^83^ Other factors influence the outcome of cCD: period of pregnancy,^74^ ^,^ ^84^ ^,^ ^85^ previous transmission and higher transmission rate,^86^ geographical origin of the mother or parasite strain,^84^ ^,^ ^87^ virulence of infecting isolate, genetic regulation of immune response of infant and mother, malnutrition, poverty and cesarean delivery avoiding gut colonisation.

*Parasite diversity* - In cohorts of infected mother from Chile, Southern Brazil, and Paraguay and Argentina registered TcI, TcII, TcIII, and TcVI lineages were usually related to the predominant regional lineage and TcV in Bolivia was associated with high cCD transmission rate.^88^ ^,^ ^89^ ^,^ ^90^ In Argentina, Honduras and Mexico, non TcI predominates in the maternal samples analysed^63^ while in Peru and Mexico, TcI like genotype was observed in a few samples.^91^ ^,^ ^92^ In Argentina identity between most TcIId lineages predominates in the mother/neonate pairs,^88^ however, minor variants suggest the presence of different TcIId variants or selection at placental level and/or neonates immune response.^88^ ^,^ ^89^ ^,^ ^93^ ^,^ ^94^

*T cruzi virulence factors* - *T cruzi* strains present different abilities to cause placental infection.^87^ The sequence of the protease TcGP63, considered as a virulence factor, was analysed in parasite clones from mother/infant pairs.^94^ No congenital murine infection was observed both with *T. cruzi* K98 clone and an isolate from congenital case VD/TcVI. However, the latter induced upregulation of genes of innate immune response and IFNγ^95^ secretion in placenta. Comparing isolates, VC/TcVI was more infective in human trophoblast than Y strain/TcII.^96^ Moreover, the human isolate VC/TcVI has a higher survival rate in placenta than Tulahuén strain but both parasites are virulent in placental explants when a high inoculum is employed.^97^

**Host parasite interaction in cCD**

*Immune response in mother/foetus* - IFNγ has been reported as a key mediator to control *T. cruzi* infection,^98^ in synergism with TNF Fα by killing the parasite through nitrite oxide secretion.^99^ Impaired immune response to control the parasite was shown in infected children^´^s maternal cells compared to mothers of uninfected children. In the first group, lower levels of both TNFα and TGFβ,^100^ ^,^ ^101^ increased IL10 levels,^102^ lower IFNγ and TNFα secretion under antigen stimulation and low activation of T cell phenotype were reported.^103^ Moreover, the mother of uninfected children with parasitaemia have shown increased levels of IFNγ and TNFα in placental, peripheral and cord blood compared to infected mother without parasitaemia.^104^

On the other hand, upregulation of infected maternal cells and their respective uninfected neonates is represented by proinflammatory and anti-inflammatory cytokines (IFNγ, IL-2, and IL-4) under mitogens and/or parasite stimulation.^105^

In contrast, in infected infant, lower levels of IFNγ, decreased activity of cord blood natural killer and cord blood CD8+T cells, higher spontaneous T cell apoptosis^106^ were reported compared to uninfected newborn from uninfected mother.^107^ Moreover, infected infants show a Th1 immune response to vaccinal antigens.^108^ Finally, before the diagnosis of cCD, in the absence of IFNγ, IL17 seems to represent neonate immune response to control *T. cruzi* parasitaemia, before the diagnosis of cCD.^106^

These data suggest that a high parasitaemia plus a strong inflammatory cytokine response is associated with absence of congenital transmission. Moreover, babies from infected mother are protected from cCD by upregulation of mother immune response through innate and adaptive immunity, decreasing the chance of CD transmission.

**Approach, prevention and elimination**

Although no studies have been analysed during pregnancy, antiparasitic treatment is not recommended since teratogenic risks of benznidazole and nifurtimox have been reported in peripheral blood lymphocytes of patients exposed to the drugs,^109^ ^,^ ^110^ although no studies have been reported during pregnancy.

The elimination of cCD is the goal established by WHO after reports of more than two hundred pregnant women treated before pregnancy in Argentina and Bolivia^56^ ^,^ ^57^ ^,^ ^58^ ^,^ ^59^ ^,^ ^60^ without cCD transmission. Along with this evidence, 100% of cure was registered in infected newborn treated in the first year of life^60^ and over 95% of cure have been reported in 10 days-19 years old children (median 6.9 years), using PCR as therapeutic control at 1 and 3 years.^58^ A randomised study in Brazil enrolled older children (7-19 years) and, applying serology as therapeutic control, showed 64.7-84.7%, respectively, by intention-to treat and by per protocol analysis.^111^ In addition, in regions where other *T cruzi* lineages occur and failure rates have been reported in children, future studies with larger number of women are needed to know the incidence of cCD in treated women.^112^

Considering these findings, the treatment of 15-44-year-old women in reproductive age is strongly recommended. Table I depicts their estimated number in countries of endemicity and Table II shows the estimates of pregnant women in countries of non-endemicity who should be screened before the pregnancy to receive antiparasitic treatment.

In Brazil, the recent approval of the compulsory notification of the chronic CD in Portaria No. 264 of February 17, 2020, will contribute for the control of code. In parallel, a PAHO initiative represents an excellent strategy in endemic regions to eliminate maternal and child infection caused by HIV, syphilis, hepatitis B, and Chagas’ disease.^113^

Although no country of non-endemicity has a national policy for screening to control congenital transmission, some regions in Spain, Italy and Switzerland implemented CD screening during antenatal care and newborns follow up for diagnosis and treatment of cCD. In Australia and New Zealand, interventions have been considered to identify pregnant women at risk of transmission.

Moreover, cost savings analyses showed that this strategy in areas of non-endemicity represents the best strategy for cCD control.^114^ ^,^ ^115^ ^,^ ^116^

*Comments* - In conclusion, cCD elimination depends on surveillance for diagnosis of infected women in reproductive age, pregnant infected women and infected babies. A new tool not accessible in the routine diagnosis worldwide is qualitative PCR, necessary for early diagnosis of cCD associated to reliable serological tests. Finally, cCD elimination around 2030, as proposed by WHO, requests joining government and Community efforts, to support a strong primary health and antenatal care to guarantee antiparasitic treatment of infected children and infected reproductive age women to achieve this goal.

**Oral transmission**

In the context of major prevalence of millions of chronic cases of CD in Latin America, acute orally transmitted cases emerged as outbreaks in Amazon extending to South American Andean and coast mountain areas in Brazil, Venezuela, Colombia, Bolivia and French Guiana.^117^ ^-^ ^124^ Interestingly, these outbreaks were registered in non- endemic areas in Brazil, where intradomicile and peri domestic triatomine were under vector control.^20^ ^,^ ^24^

During 1965-2009, 138 outbreaks were reported, predominantly in Brazilian Amazon while only 7-8 occurred in areas outside Amazonia. Between 2000-2009, 855 cases represent an increased number of ACD.^125^ Approximately 3.060 ACD cases were reported in Brazil, between 2007-2019, predominantly in North region (94.4%) and Pará (74.54%), the majority attributed to the ingestion of contaminated food.^126^ Temporal comparison shows a trend towards an increasing incidence coefficient of oral transmission in the last 4 years, when a total > 300 cases /year were recorded.^126^ ^) I^n addition, in Venezuela from 2011-2015, 11 outbreaks involved 249 people, predominantly children,^122^ and in Colombia from 1999-2017, 18 outbreaks affected 576 people.^118^ ^,^ ^123^

**Risks factor for oral transmission of CD**

*Changes in sylvatic cycles* - Deforestation introduces changes in wildlife biodiversity and in sylvatic cycles of vectors and animals.^127^ Human activities exploring açaí as a source of food and economic survival closer to the sylvatic cycle and forest have been suggested as a risk factor for oral transmission of CD.

On the other hand, in Amazon and some Andean regions, several palm trees distributed throughout Colombia, Ecuador, Guatemala, Mexico, Panama, Peru, Venezuela and Brazil have been reported as ecotopes for a broad range of triatomine vectors. High vector infection rates make possible the contamination of homemade food or beverages.^17^ ^,^ ^122^ ^,^ ^123^ ^,^ ^124^ Other source of infection is represented by sylvatic hosts, especially *Didelphis*, found in deforested areas near the outbreaks with infectant parasite forms in 12-100% of their anal glands secretion.^123^ ^,^ ^128^ ^,^ ^129^

*Lack of good practices in food preparation* - Table V represents the outbreaks with the possible source of contaminated food (açaí or bacaba fruit), water or soup, juices, water or soup, guava, orange, tangerine juices, mayo fruit juice or “viño de palma”, shared by the people involved in the outbreaks,^118^ ^,^ ^119^ ^,^ ^120^ ^,^ ^121^ ^,^ ^124^ ^,^ ^129^ ^-^ ^140^ contaminated with infected triatomine or their feces; or with anal glands secretions of infected marsupials.

**TABLE V:** Orally transmitted acute Chagas disease: number of people possibly involved, suspected food, triatomines reservoirs, and cause of death^*a*^

Region urban/rural	Suspected food	No †/Total	Infected triatomines	Infected reservoirs	Cause of death	First author, year
Teutônia-Rio Grande do Sul, BR, rural, 1965	Shared meal	6/17	*T. megistus*	*D. marsupialis*	Acute myocarditis	Silva^(130)^ 1968
Belém, Pará, BR, urban, 1968	Shared meal	1/4	NR^*b*^	NR	Sudden death	Shaw^(131)^ 1969
Catolé do Rocha, Paraíba, BR, rural, 1986	Shared sugar cane juice	1/26^*c*^	Triatomine: peri-domicile	*D. albiventris*	Myocarditis	Shikanai-Yasuda^(129)^ 1991
Abaetetuba-Pará, BR, periurban, 1998	Shared açai paste/juice	0/13	NR	NR	NR	Pinto^(132)^ 2001
Navegantes, Santa Catarina, BR, periurban,2005	Sugar cane juice	3/25	*T. tibiamaculata*	Opossums	Heart failure; digestive bleeding	Health Ministry Brazil^(123)^ 2005
Pará, BR, urban/rural,1988-2005	Açaí, regional fruits paste/juice	2/12 ?^*d*^ /181	Triatomines peridomicile	NR for outbreaks	Myocarditis, digestive bleeding	Pinto^(133)^ 2008
Breves-Bagre, Pará, BR, urban, 2007	Shared food	0/27	Not found	NR	NR	Beltrão^(134)^ 2009
Macaúbas, Bahia, BR, small town, 2006	Shared water or beverage	2/7	*T. sordida*	Opossums	Heart failure	Dias^(135)^ 2008
Barcarena, Pará, urban, 2006	Shared açai paste/juice	0/11	Not found 90 days later	NR	NR	Nobrega^(136)^ 2009
Redenção, Ceará, BR, rural, 2006	Shared soup with fresh herbs	0/8	*T. brasiliensis*, *P. lutzi:* no infected after pesticides	marsupials/rodents	NR	Cavalcanti^(137)^2009
Lebrija, Santander Colômbia, 2008	Shared orange or tangerine juice?	2/10	No infected *P. geniculatus*	marsupials No infected	Acute myocarditis	Hernandez^(118)^ 2009
Caracas-Venezuela, urban, 2007	Shared guava juice	1/103^*e*^	Triatomine	No infected rodent	Acute myocarditis	Noya^(119)^2010
Ibipitanga, Bahia, BR, rural, 2010	Sugar cane juice	0/6	*Triatoma sordida*	NR	NR	Bastos^(138)^2010
Marcelino Vieira, Rio Grande do Norte, BR, rural, 2016	Sugar cane juice	3/18	*T. brasiliensis/ T. tibiamaculata*	NR	NR	Vargas^(139)^ 2018
Lábrea, Amazonas, BR, small town, 2017^(139)^	Açaí pulp	NR/10	NR	NR	NR	Santana^(140)^ 2019

†: deaths; *a*: modified from Shikanai-Yasuda and Carvalho.^(20)^; *b*: NR - no report; *c*: a second febrile patient died of unknown cause. *d*: death rate of cases transmitted orally has not been described; *e*: total number unknown (no information on Rheumatoid Factor absorption for IgM anti-*T. cruzi* test).

**Clinical symptoms**

Oral transmission of CD is considered when > 1 acute case of febrile disease without other causes is linked to a suspected food and should be confirmed by the presence of the parasite in the patients´ blood or biological fluid sample and/or suspicious food by direct microscopic examination.

Incubation period is 3-22 days for oral transmission 38-39ºC and the involvement of phagocytic mononuclear system with splenomegaly, hepatomegaly, adenomegaly^20^ ^,^ ^119^ ^,^ ^129^ ^,^ ^132^ ^,^ ^133^ ^,^ ^134^ ^,^ ^135^ ^,^ ^138^ and peri palpebral oedema. However, some peculiarities need to be emphasised. No signs of parasite entry like Romaña signal are present, but facial oedema particularly peri palpebral oedema is very frequent, exanthema (maculopapular, petechial or erythema nodosum) and cardiac manifestations (pericardial effusion, pleural effusion, and icterus), are more commonly seen in oral rather than vector-borne disease. Gastric haemorrhage possibly represents entry through the digestive mucosa, which shows amastigotes in an intense inflammatory infiltrate.^141^

*Definition of ACD orally transmitted* - Laboratorial definition of ACD by oral transmission in oligosymptomatic patients represents a challenge when direct microscopy was not positive due to late suspicion or lack of application of concentration methods. In these cases, patients with unrecognised chronic CD and febrile disease are at risk of ACD diagnosis if only indirect enrichment parasitological methods, qualitative PCR, and even serology (without increasing titers) or false positive IgM^142^ are considered. As such parasitological and molecular tests are positive in chronic CD,^143^ they are useful only in previously non infected patients with acute symptoms and epidemiological link. Quantitative PCR could be useful if high DNA parasite counts not present in chronic cases were observed.^144^ In addition, live parasites found in the suspect food for the first time, confirmed the proved disease by oral contamination.^140^ Therefore, the new advances in molecular qualitative and quantitative methods are challenges to be included in the classic concepts of confirmed, probable and suspected cases (PAHO),^145^ as shown in the Table VI.

**TABLE VI:** Proposed criteria for case definition in oral transmission of Chagas disease^*(145)a,b*^

Case definition	Micro haematocrit, strout, buffy coat fresh exams, stained smears, and thick drop^*c*^	More than 1 case	PCR^*a*^	Immunological criteria IgG increase/IgM^*d*^	Clinical features without chagoma	Parasite in the food^*b*^	Relation with contaminated food	Other causes	Link to other case
Confirmed	+ direct microscopy	X	X	X	X	X	X	No	X
Probable	X	X	X	X	X	No	-	No	X
Suspected	No	X	-	-	X	No	-	No	X

*a*: presence of DNA is considered similarly to positivity of indirect parasitological methods in previously non immune (non infected patient. As DNA copies of fragments and enrichment parasitological methods are positive in chronic cases, a positive result of qualitative polymerase chain reaction (PCR) is valid for confirmation of only previously non infected cases. Quantitative PCR with a high number of copies not seen in chronic cases is more helpful to distinguish acute Chagas disease (ACD) from chronic CD; *b*: presence of live parasite in the suspected food by any method fills the criteria for proved case c and was firstly described by Santana et al;^(140)^ *c*: concentration methods (strout, microhematocrit and buffy coat) are more sensitive than fresh exams or stained smears; *d*: IgM antibody is not reliable d due to the lack of accessible reliable reagents, particularly reliable positive controls, and the presence of false positive results due to IgM anti IgG antibodies not easily detectable in commercial kits.

**Challenges**

*Pathogenesis and parasite strain* - Analyses of Tc lineage in patients, reservoir and vectors possibly involved in ACD outbreaks are useful to identify possible sources of contamination.^146^ In addition, several lineages showed differential virulence regarding the route of infection in experimental infection. So, Peruvian strain (TcII) is less virulent by gastric than intraperitoneal inoculation whereas Colombian strain (TcI, sylvatic cycle), infects by gastric as well as by intraperitoneal route.^147^ Metacyclic forms of gp82-expressing Y82 strain (related to TcVI human outbreaks) have been reported as better adapted to invade gastric mucosa and to cause oral infection than Y30 strain (TcII).^148^ Moreover, TcIV orally infected mice (with both reference and isolated strains from human oral outbreak) showed higher parasitaemia and tissular parasitical load than those intraperitonially infected.^149^ ^,^ ^150^ In addition, TcI was less infective by oral route than TcIV which is less infective than TcVI. As TcIV express less pepsin-resistant gp-90 (which downregulated cell invasion) than TcI, their invasion capacity in gastric epithelium is higher than TcI.^151^ In summary, the severity of the infection depends on parasite factors (load, glycoprotein resistant to gastric juice), and host factors (gastric secretions, regulation of invasion process and hosts immune response). At oral level, Th2 immune response protects the host, however a Th1 immune response is necessary to protect the host against systemic infection.^152^

*Inactivation of the parasite in the food* - Considered since 1921 as the natural route of vectors and animals contaminations and a mechanism for parasite dispersion among animals,^153^ orally transmitted human infection was first documented in 1936.^154^ In addition, sylvatic and domestic animals have been experimentally infected by food contaminated with triatomines or their feces.^155^ ^,^ ^156^ Parasites survival in contaminated fruits and vegetables experimentally contaminated with *T. cruzi* was reported from 6 to 72 h,^157^ in sugar cane up to 12 h by direct methods and up to 24 h by experimental inoculation^158^ and are extremely sensitive to dryness, although resistant to extremes of pH and temperature. In mice virulence has been demonstrated after frozen at -20ºC for up to 26 h.^159^ Inactivation of Tc in açaí pulp has been shown by heating above 45ºC and pasteurisation.^160^ Heating fruits (70 ± 1ºC for 10 s) or pasteurising juice (82.5 ºC for 1 min) inactivates the parasite. In addition, *T*. *cruzi* I and *T*. *cruzi* III have been reported to be more resistant to chemical products like sodium hypochlorite and heat temperature compared to Y strain.^161^

*Molecular methods to search for parasites in the food* - Improvement of methods to detect parasites were reported with detection of DNA copies in 10% of commercialised açaí derived products with mixtures of Tc I-TcII and more rarely TcIV and TcVI.^162^ In addition, quantitative PCR^163^ ^,^ ^164^ proved to be sensitive to search parasite in the food. Recently, possible detection of live parasites in food by mRNA-base reverse transcriptase PCR could represent a new strategy to ensure a better-quality food and to improve handmade and manufactured food.^161^

*Comments* - The dramatic increase in the outbreaks of orally transmitted CD seen in the last 12 years and mainly in the latter years is likely to be attributed to the lack of good handling practices in food processing, and changes in sylvatic cycles, and to the better recognition of this disease. Although the Brazilian Health Minister and Programa Estadual da Qualidade do Açaí, Pará government have recommended good handling practices to control food contamination,^165^ ^,^ ^166^ ^,^ ^167^ unfortunately, evidences of lack of good quality of food were shown even with general impurity, microbes and parasite DNA presence in commercially available açaí products.^168^ ^,^ ^169^ ^,^ ^170^

Since açaí is employed as food and as source of subsistence by Amazon population, policies for good practices in food preparation should be monitored by rigorous health surveillance, including food heating above 45ºC and/or pasteurisation,^160^ ^,^ ^167^ associated to the application of new tools for detection of parasite DNA or mRNA for food quality control.^161^ ^,^ ^162^ ^,^ ^163^ ^,^ ^164^

On the other hand, as access for early diagnosis and treatment is not easy in Brazilian Amazon, active surveillance on ACD cases should be implemented by improvement of the structure for diagnosis as well as for health attention to severe cases of ACD, including the search of ACD in Febrile Syndrome Surveillance and training of health professionals for diagnosis and management of orally transmitted CD.
