# ERRATUM

**DOI:** 10.1590/0074-02760210033er

**Published:** 2024-02-09

In the article “**Emerging and reemerging forms of *Trypanosoma cruzi* transmission**”, DOI number: 10.1590/0074-02760210033, published in *Mem Inst Oswaldo Cruz*, Rio de Janeiro, Vol. 117: e210033, 2022, on page 6 (Figure):

Where it reads:

2. PCR quali (non immune recipient) or quantitative - before the transplant, once a month, first month

It should read:

2. PCR quali (non immune recipient) or quantitative - before the transplant, once a **week**, first month

